# Necrotizing fasciitis of the extremities: a prospective study

**DOI:** 10.1007/s11751-011-0116-1

**Published:** 2011-08-24

**Authors:** Ramin Espandar, Siamak Yousef Sibdari, Elham Rafiee, Shideh Yazdanian

**Affiliations:** 1 Orthopedic Surgery Department, Tehran University of Medical Sciences, Imam Khomeini Hospital Complex, Keshavarz Blvd., 1419733141 Tehran, Iran; 2 Pediatrics Department, Tehran University of Medical Sciences, Imam Khomeini Hospital Complex, Tehran, Iran; 3 Dermatology Department, Tehran University of Medical Sciences, Imam Khomeini Hospital Complex, Tehran, Iran

**Keywords:** Necrotizing fasciitis, Skin diseases, bacterial, Musculoskeletal disease, fasciitis

## Abstract

Necrotizing fasciitis is a rapidly progressive infection and is a necrosis of the fascia and surrounding tissues. Despite recent advances in its management, outcomes have not improved and mortality rate is still high. Between September 2007 and August 2009, we prospectively studied twenty-four histopathologically proven necrotizing fasciitis patients to assess the prognostic factors that indicate the outcome. Mortality rate was 20.8%. Twelve patients (50%) improved, while seven patients (29.2%) were complicated by limb loss. Mortality rates related to upper and lower limb involvement were similar (20% vs. 22.2%). The rates of gangrene and amputation in patients with diabetes mellitus were significantly higher than other comorbidities. Patients with gram-positive infections had significantly lower rates of amputation (15.4% vs. 54.5%, *P* = 0.04). Mean band cell count and serum potassium level were significantly higher in the nonsurvivors same as leukocyte count in the patients with gangrene, while serum sodium level was significantly lower in nonsurvivors. We conclude that hyponatremia, hyperkalemia, and increased band cells in the peripheral blood of patients may be useful parameters in distinguishing life-threatening necrotizing fasciitis; hence, we recommended lower threshold to amputation during surgery for this group of patients.

## Introduction

Necrotizing fasciitis is an uncommon soft tissue infection characterized by extensive rapidly progressive necrosis of the fascia and subcutaneous tissues. It has a high rate of mortality ranging from 6 to 76%. Clinical presentations can be indolent or fulminant [1]. Risk factors include diabetes mellitus, advanced age, obesity, liver disease, malignancy, alcoholism, or other immunosuppressive disorders. In recent years, an increased incidence has been attributed to the intravenous drug abuse [2]. Initial signs and symptoms are nonspecific including erythema, pain, edema, fever, crepitus, and induration [3]. However, the differential diagnosis has a wide spectrum. The bacterial etiology is often polymicrobial. The most common organism is Group A streptococcus, which can implicated in otherwise healthy individuals. Other microorganisms include staphylococcus, peptostreptococcus, bacteroid, and clostridium [4, 5]. In spite of recent progress in the treatment methods, the outcome has not improved and mortality rate is still high. Orthopedic surgeons are often involved in the management of patients with necrotizing fasciitis; hence, they should be aware of the presentation and management of this life-threatening condition.

The purpose of the present study was to evaluate diagnostic, therapeutic, and prognostic aspects of the necrotizing fasciitis of the extremities.

## Materials and methods

After receiving institutional review board approval and informed consent from individual patients, 24 consecutive patients who were managed for necrotizing fasciitis of the extremities were recruited to this study. Treatment was provided at a university hospital between September 2007 and August 2009.

There were 27 cases with primary diagnosis of necrotizing fasciitis, but three of them were not proven histopathologically; hence, these cases were excluded from the study. The study group consisted of consecutive patients with necrotizing fasciitis of the extremities that was diagnosed clinically and confirmed with histopathologic study. Management of the patients was performed according to the existing standard protocols. Primary diagnosis was made according to the clinical characteristics of necrotizing fasciitis (Table 1) [4]. Empirical antibiotic therapy was administered according to the recommendation of a specialist in infectious diseases, which might be changed later on the outcome of culture results. Debridement was performed as soon as possible. Surgical exploration was carried out for suspicious cases for both diagnostic and therapeutic purposes. During exploration, the presence of fascial necrosis and myonecrosis (dusky gray subcutaneous fat and fascia with a scanty serosanguineous discharge), lack of resistance to blunt dissection of the normally adherent superficial fascia, lack of bleeding, and the presence of foul-smelling pus were indicative of necrotizing infection. All necrotic tissues were debrided, and culture obtained from the area of worst affected. All affected components including subcutaneous tissue, fascia, and muscles were removed and sent for histopathologic diagnosis of necrotizing fasciitis to include in the study. The wound was left open, and the patient was taken to the operating room again for inspection and further debridement every 24–48 h depending on the appearance of the wound. After removing all necrotic tissues and when the infection was under control with a healthy granulating tissue growth, wound closure with direct suture, skin graft, or flaps was done. Tissue biopsy specimen was prepared for all of the patients. Amputation was performed for the patients who had ascending infections not being controlled by repeated debridement, exposed joints after initial debridement, and severe sepsis. Early amputations in these patients were preferred to control the septic foci in order to reduce the number of surgical debridements and consequently, the risks associated with each anesthetic procedure. Data were collected through a questionnaire and include demographics, associated diseases, culture results, laboratory studies, received treatments, and final outcome. Only histopathologically confirmed cases (all of 24 patients) were included in the study. The patients were followed for the outcome including improvement, limb loss, or death. Improvement was defined as removal of necrotic tissue, control of infection, and proper soft tissue coverage of the affected limb that led to a viable limb. Survivors were the patients who recovered from the disease either with improvement or after amputation, whereas nonsurvivors were the patients who died as a result of the disease. Data analysis was performed using SPSS software version 11.5. The statistical methods used to compare groups were chi-square test and *t*-test (independent samples test).

**Table 1:** Clinical characteristics of necrotizing fasciitis

Stage	Features
I (early)	Erythema, swelling, warmth, tenderness, fever
II (intermediate)	Blister and bullae, skin fluctuance, skin induration
III (late)	Hemorrhagic bullae, skin hyposensitivity or anesthesia, crepitation, tissue necrosis progressing to gangrene

Wong and Wang [17]

## Results

Twenty-four patients (18 men and 6 women) with histopathologically proven diagnosis of necrotizing fasciitis of the extremities were included in the study. The mean age (±SD) was 49.5 ± 16.1 years. Fifteen patients had lower limb infection, and others had upper limb infection. Median time from the appearance of symptoms to the diagnosis was 15 days (interquartile range: 7–27.25) and from the primary diagnosis to the surgical treatment was 1.5 days (interquartile range: 0–3).The difference between survivors (either improved or amputated patients) and nonsurvivors regarding the time from primary diagnosis to surgical intervention was not statistically significant. Twenty patients (83.3%) had fever, and two patients (8.3%) presented with confusion.

**Fig. 1:**
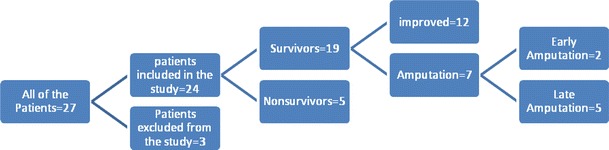
Outcome of the patients

Table 2 shows local findings at the site of infection in our patients. Comorbidities are listed in Table 3. Twelve patients (50%) improved, seven patients (29.2%) were complicated by limb loss and none of them died (in two patients, amputation was done during the first surgical debridement and in five patients during later debridement), and 5 patients (20.8%) died during management of the disease (Fig. 1). There was no significant relation between the mortality rate and local symptoms of the patients.

**Table 2:** Clinical characteristics of the infection site

Variable	No. of cases (%)	Survivors (%)	Nonsurvivors (%)
Warmth	19 (79.2)	11 (57.9)	5 (100)
Erythema	16 (66.7)	8 (42.1)	5 (100)
Gangrene	9 (37.5)	7 (36.8)	1 (20)
Discharge	7 (29.2)	4 (21)	2 (40)

There were no statistically significant difference between survivors (*N* = 19) and nonsurvivors (*N* = 5)

**Table 3:** Incidence of preexisting conditions

Comorbidity	No. of cases (%)	Survivors (%)	Nonsurvivors (%)
Smoking	15 (62.5)	10 (52.6)	2 (40)
IV drug usage	11 (45.8)	7 (36.8)	2 (40)
DM	7 (29.2)	5 (26.3)	1 (20)
Renal disease	2 (8.3)	1 (5.2)	1 (20)
Malignancy	4 (16.7)	2 (10.5)	2 (40)
Peripheral vascular disease	1(4.2)	1 (5.2)	0
HIV infection	4 (16.7)	2 (10.5)	2 (40)
Immunosuppression	3 (12.5)	2 (10.5)	0

There were no statistically significant difference between survivors (*N* = 19) and nonsurvivors (*N* = 5)

The rates of mortality following the upper and lower limb involvement were similar (22.2% vs. 20%, *P* > 0.05), whereas the lower limb infection had the higher rate of amputation, in which this difference was statistically significant (40% vs. 11.1%).

**Graph 1:**
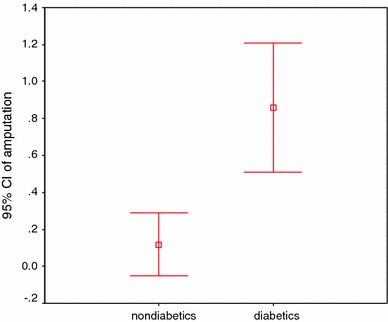
Rate of amputation in patients according to diabetes mellitus

Rate of gangrene was significantly higher in patients with diabetes mellitus (71.4% for those who had diabetes mellitus vs. 23.5% for others, *P* = 0.02), but we found no association between gangrene and other comorbidities. The rate of limb loss was also significantly higher in diabetic cases [71.4% of diabetic patients suffered from amputation vs. 15.4% of nondiabetics (*P* = 0.012) (Graph 1)]. Improvement in diabetic patients was significantly lower than in nondiabetics (14.3% vs. 64.7%), (*P* > 0.025).

The results of tissue cultures are listed in Table 4. Mortality rate in patients affected by gram-positive cocci and other organisms was 30.8 and 9.1%, respectively, but this difference was not statistically significant (*P* = 0.1). Patients with gram-positive infections had significantly lower amputation rate (15.4% vs. 54.5%, *P* = 0.04).

**Table 4:** Tissue culture results

Culture result	No. of cases (%)	Survivors (%)	Nonsurvivors (%)
Acinetobacter	3 (12.5)	2 (10.5)	0
Enterobacter	3 (12.5)	7 (36.8)	4 (80)
Gram-positive cocci	13 (54.1)	3 (15.7)	0
Polymicrobial	5 (20.8)	3 (15.7)	1 (20)

There were no statistically significant difference between survivors (*N* = 19) and nonsurvivors (*N* = 5)

Culture results were positive for gram-positive cocci in 14.3% diabetic patients versus 70.6% nondiabetics (*P* = 0.012).

Mean (±SD) age of survivors and nonsurvivors was 55.4 ± 15.6 and 41 ± 13.8 years, respectively (95% CI of the difference (−2.1 to 31), *P* = 0.08). Mortality rate was not associated with sex (20% in women vs. 26.7% in men, *P* > 0.05). Additionally, there was no association between the amputation and mortality rates.

The mean (±SD) age was 56 ± 20.2 years in patients with gangrene of the extremities versus 45.7 ± 12.3 years in other patients (mean difference = 10.2, 95% CI of the difference (−3.4 to 23.9), *P* = 0.1).

Laboratory findings on hospital admission are shown in Table 5. Mean band cell count and mean serum potassium level was higher in nonsurvivors compared with survivors on hospital admission and this was statistically significant (68.3% vs. 24%, *P* = 0.08 and 4.7 meq/l vs. 3.9 meq/l, *P* = 0.03 respectively) at the time of admission (127.7 meq/l vs. 134.3 meq/l *P* = 0.008). Moreover, mean WBC count was highest in the patients admitted with gangrene of the limb (20 × 10^3^/μl vs. 14 × 10^3^/μl) which was statistically significant (*P* = 0.02).

**Table 5:** Mean (±SD) of laboratory findings on hospital admission

Variable	Overall	Survivors	Nonsurvivors	Normal
WBC count × 10^3^/mm^3^	16.29 ± 6.3	15.8 ± 6.2	20.2 ± 7.9	4.5–11
Band cell (%)*	32 ± 27.6	24 ± 19.5	68.3 ± 29	3–5
Platelet × 10^3^/mm^3^	230.2 ± 112.2	233.4 ± 119	175.8 ± 88	150–400
PT (s)	16 ± 1.8	16 ± 1.8	16 ± 1.6	11–13.5
PTT (s)	36.5 ± 8.8	37 ± 9.5	40 ± 5	25–35
Serum protein (g/dl)	4.7 ± 0.58	4.8 ± 0.5	4.4 ± 0.5	6–8
Serum albumin (g/dl)	3.1 ± 0.66	3.3 ± 0.39	3.3 ± 0.58	3.5–5.5
Alk-p (IU/l)	228.8 ± 146.1	202 ± 144	269 ± 115	35–150
ALT (IU/l)	52.5 ± 38.9	42.6 ± 14.3	40.2 ± 20.6	1–45
AST (IU/l)	56.1 ± 29.4	50.7 ± 21.6	50 ± 25.6	1–36
BUN (mg/dl)	47.5 ± 22.2	42.9 ± 18.9	55.5 ± 28.3	11–23
Cr (mg/dl)	1.13 ± 0.37	1.1 ± 0.2	1.3 ± 0.69	0.6–1.2
Sodium (meq/l)*	132.8 ± 3.3	134 ± 2.4	127.7 ± 2.6	135–145
Potassium (meq/l)*	4 ± 0.63	3.9 ± 0.54	4.7 ± 0.7	3.5–5
Calcium (mg/dl)	8.8 ± 0.7	8.9 ± 0.4	9.1 ± 1.1	8.4–10.6
Blood pH	7.41 ± 0.07	7.4 ± 0.04	7.38 ± 0.12	7.35–7.45
Bicarbonate (meq/l)	21.9 ± 4.3	22.2 ± 3	17.6 ± 4.8	21–27

*WBC* white blood cell; *PT* prothrombin time; *PTT* partial thromboplastin time; *AST* aspartate aminotransferase; *ALT* alanine aminotransferase; *BUN* blood urea nitrogen

*P value < 0.05

## Discussion

Necrotizing fasciitis was first described in 1848. The incidence has been reported in 0.4–0.53 cases per 100,000 in the United States [4, 5]. There may be an increased incidence in Asian and African countries. A high index of suspicion is required for the diagnosis of necrotizing fasciitis. The patient may present only with pain, erythema, and some swelling, so the disease is often mistaken as cellulitis. Necrotizing fasciitis can often progress within a few hours, and rapid progression of the erythema and induration in spite of treatment with broad-spectrum antibiotic is an important diagnostic clue [4].

Anaya et al. [6] estimated that extremities were the most common site of necrotizing fasciitis in 57.8% followed by the abdomen and perineum. It is an invasive and destructive infection that complicates the skin, subcutaneous tissue, deep fascia, and, to some extent, sparing of muscle. General signs, such as fever and severe systemic reactions, may be present.

Intramuscular injections and intravenous (IV) infusions may lead to necrotizing fasciitis. In our series, 45.8% of the cases were IV drug abusers. Angoules et al. [7] also addressed drug abuse and needle punctures in the affected site as the commonest cause of necrotizing fasciitis of extremity, and drug abuse and needle punctures were found in 129 (33%) patients in a systematic review of necrotizing fasciitis of upper and lower limb.

Gonzalez et al. [8] have also reported a higher amputation rate in patients with polymicrobial, anaerobic, and gram-negative infections. In our series, patients with gram-positive infections had significantly lower amputation rates. It seems that higher amputation rate in diabetic patients may be related to different organisms such as anaerobics or gram-negative infections.

Necrotizing fasciitis can be difficult to recognize in early stages, especially in patients with diabetes mellitus. In this series, mean delay to diagnosis was considerably higher in diabetic patients.

Although in our series delayed diagnosis or treatment was not significantly related to mortality rate, in some studies, early diagnosis and immediate, radical surgery improved the survival rate [9, 10].

Ogilvie and Miclau [11] showed that elevated serum potassium is a relative mortality risk factor (relative risk (95% CI) = 5.40 (1.90–15.3)) and mean serum potassium in nonsurvivors and survivors was 4.6 mmol/l versus 3.9 mmol/l, respectively. In our patients, same results for mean serum potassium were found (4.7 in nonsurvivors vs. 3.9 in survivors). In addition, in our series, mean serum sodium was significantly lower in nonsurvivors (mean: 127.7). These findings may represent renal dysfunction due to multiorgan damage caused by more severe infection and also hyponatremia that may be due to fluid sequestration in more severe soft tissue infections. So hyponatremia and hyperkalemia may be useful parameters in distinguishing life-threatening necrotizing fasciitis, which may suggest more aggressive management in these groups of patients.

Hsieh compared platelet counts in patients with necrotizing fasciitis and in patients with cellulitis and found lower platelet counts in the first group (mean: 194.0 × 10^9^/l vs. 299.3 × 10^9^/l *P* = 0.03) [12], but such relationship has not been found in our study.

According to the literature, mortality rate resulting from necrotizing fasciitis ranges from 9.3 to 76% [13]. Callahan et al. [14] performed a meta-analysis of 14 studies and assigned an overall mortality rate of 26%. Pessa and Howard [15] also demonstrated a mortality rate of 18% in extremity infection compared with 44 and 33% in abdominal and perineal infections, respectively. In our series, the overall mortality rate was 20.8%, and mortality rates in patients with upper and lower limb infection were 22.2 and 20%, respectively. In our series, the risk of amputation was significantly higher in lower limb involvement, but survival rate in upper or lower limb involvement was not significantly different.

Aggressive treatment of the patient with suspected necrotizing fasciitis can reduce morbidity and mortality rate [16]. Treatment should be initiated as soon as a necrotizing soft tissue infection is suspected and should include broad-spectrum antibiotics, resuscitation, early debridement, and early second surgical exploration [8]. In our series, we did early amputation as the first surgical intervention in two cases, which was life saving for them. This study can highlight the importance of early recognition of mortality risk factors and decision for early amputation as a life saving surgical procedure in this group of patients with necrotizing fasciitis.

According to our findings at the time of admission, the presence of hyponatremia, hyperkalemia, and increased band cell count in peripheral blood are risk factors for mortality, and the presence of diabetes mellitus increased the risk of amputation in lower limbs. So, lower threshold to amputation may be mandatory in this group of patients.
